# Differentiation of Transformed Bipolar Disorder From Unipolar Depression by Resting-State Functional Connectivity Within Reward Circuit

**DOI:** 10.3389/fpsyg.2018.02586

**Published:** 2018-12-21

**Authors:** Jiabo Shi, Jiting Geng, Rui Yan, Xiaoxue Liu, Yu Chen, Rongxin Zhu, Xinyi Wang, Junneng Shao, Kun Bi, Ming Xiao, Zhijian Yao, Qing Lu

**Affiliations:** ^1^Department of Psychiatry, The Affiliated Nanjing Brain Hospital of Nanjing Medical University, Nanjing Medical University, Nanjing, China; ^2^School of Biological Sciences and Medical Engineering, Southeast University, Nanjing, China; ^3^Key Laboratory of Child Development and Learning Science, Southeast University, Nanjing, China; ^4^Nanjing Medical University, Nanjing, China

**Keywords:** bipolar disorder, depression, reward circuit, functional connectivity, resting-state, functional magnetic resonance imaging

## Abstract

Previous studies have found that neural functional abnormalities detected by functional magnetic resonance imaging (fMRI) in brain regions implicated in reward processing during reward tasks show promise to distinguish bipolar from unipolar depression (UD), but little is known regarding resting-state functional connectivity (rsFC) within the reward circuit. In this study, we investigated neurobiomarkers for early recognition of bipolar disorder (BD) by retrospectively comparing rsFC within the reward circuit between UD and depressed BD. Sixty-six depressed patients were enrolled, none of whom had ever experienced any manic/hypomanic episodes before baseline. Simultaneously, 40 matched healthy controls (HC) were also recruited. Neuroimaging data of each participant were obtained from resting-state fMRI scans. Some patients began to manifest bipolar disorder (tBD) during the follow-up period. All patients were retrospectively divided into two groups (33 tBD and 33 UD) according to the presence or absence of mania/hypomania in the follow-up. rsFC between key regions of the reward circuit was calculated and compared among groups. Results showed decreased rsFC between the left ventral tegmental area (VTA) and left ventral striatum (VS) in the tBD group compared with the UD group, which showed good accuracy in predicting diagnosis (tBD vs. UD) according to receiver operating characteristic (ROC) analysis. No significant different rsFC was found within the reward circuit between any patient group and HC. Our preliminary findings indicated that bipolar disorder, in early depressive stages before onset of mania/hypomania attacks, already differs from UD in the reward circuit of VTA-VS functional synchronicity at the resting state.

## Introduction

Bipolar disorder (BD) and unipolar depression (UD) are two of the most debilitating illnesses worldwide (Murray and Lopez, 1997). BD mainly differs from UD in the presence of mania/hypomania. Clinical manifestations of BD are more complex than those of UD. However, approximately half of bipolar individuals present with a major depressive episode as their first mood episode (Tondo et al., 2010; Etain et al., 2012). When in a depressive episode, symptoms are similar in BD and UD, which heavily obstructs the accurate diagnosis of BD. Up to 60% of BD patients seeking treatment for depression are initially diagnosed with UD. Only 20% BD who are experiencing a depressive episode are precisely diagnosed within the first year of treatment (Hirschfeld et al., 2003). Moreover, treatments for BD and UD are very different, with stabilizers for BD and antidepressants for UD being prescribed. Inappropriate medication might lead to poor prognosis, such as increased suicidal behavior, switching to mania, and higher health care costs (Bowden, 2010; Goodwin, 2012; Baldessarini et al., 2013). Therefore, it is of great importance to distinguish BD from UD as early as possible.

Numerous clinical characteristics have been recognized as risk factors for developing BD, including (1) family history of BD or affective disorder, (2) early age of onset (less than 25 year-old), (3) recurrence (more than 4 episodes), (4) substance abuse, (5) psychotic symptoms, and (6) refractory (Ostergaard et al., 2014; Tondo et al., 2014; Woo et al., 2015; Bukh et al., 2016; Ratheesh et al., 2017). In addition, several clinical rating scales may help to detect subthreshold manic/hypomanic symptoms in depression, such as the Hypomania Checklist (Angst et al., 2005), the Screening Assessment of Depression Polarity (Solomon et al., 2006), and the Bipolar Inventory Symptoms Scale (Bowden et al., 2007). Although helpful, the aforementioned strategies are based on phenomenological observation and depend heavily on the professionalism of clinicians. For early identification of BD from UD, objective methods are needed.

Neuroimaging techniques, especially magnetic resonance imaging (MRI) can objectively reflect the structural and functional condition of the neural system. Numerous MRI studies have provided evidence that individuals with BD could be differentiated from those with UD by abnormal gray matter volumes in several brain regions. For example, in a cross-sectional MRI study, Rive et al. (2016) found that depressed subjects with BD and UD could be classified based on the gray mater volumes of the middle frontal gyrus, parahippocampal gyrus, and the orbital part of the superior frontal gyrus. Other studies showed reduced ventral diencephalon volumes in euthymic BD vs. UD (Sacchet et al., 2015), reduced gray matter volumes in the hippocampus and the amygdala (Amy), but increased gray matter volumes in the anterior cingulate cortex (ACC) in individuals with BD relative to individuals with UD (Redlich et al., 2014). The white matter connectivity may also be useful in differentiating BD from UD. Damme et al. (2017) found that white matter connectivity between the nucleus accumbens (NAcc) and both the medial orbitofrontal cortex (mOFC) and Amy were associated with elevated mania/hypomania proneness. Regarding functional neuroimaging, substantial evidence indicates that abnormalities in brain regions implicated in reward processing during reward tasks show promise to distinguish bipolar from UD. Compared with healthy controls, UD showed reduced caudate and NAcc responses to rewards (Pizzagalli et al., 2009), and less ventral striatal activation during reward anticipation (Stoy et al., 2012). On the contrary, BD patients showed elevated striatal reactivity (Dutra et al., 2015), and increased functional connectivity between the ventral striatum (VS) and OFC (Dutra et al., 2017) across monetary and social rewards compared to the healthy controls.

The reward circuit mediates goal-directed behaviors, including emotions, motivation, and cognition. Key brain regions in the reward circuit are the ACC, the orbital prefrontal cortex (OFC), the VS, the ventral tegmental area (VTA) and the amygdala (Haber and Knutson, 2010). These brain reward regions have been assigned specific functions: the VTA–VS is the center of reward, the ACC and OFC are responsible for working memory and executive control, and the amygdala is crucial for associative fear- and reward-related memories (Russo and Nestler, 2013). In addition, other structures, including the dorsal prefrontal cortex, hippocampus, thalamus, and lateral habenular nucleus, are also important components in regulating the reward circuit. Connectivity between these areas forms a complex neural network that mediates different aspects of reward processing. Activation of the reward circuit leads to increased motivation, behavior directed toward attaining rewards, and positive emotions, or to anger when goal-striving is frustrated. Downregulation or deactivation of the reward circuit leads to decreased motivation, increased withdrawal, and emotions such as sadness and anhedonia. Reward hypersensitivity is suggested to underlie risk for manic/hypomanic symptoms (Alloy et al., 2016). Collectively, BD seems to be characterized with reward hyperactivation, while reward hypoactivation is involved in UD (Alloy et al., 2016).

However, there are some different findings in tasks based functional magnetic resonance (fMRI) studies. Foti et al. (2014) reported that reward processing during a laboratory gambling task was heterogeneous within MDD, indicating that not all MDD were characterized by reward dysfunction. In a study using a card-guessing task, BD patients showed decreased, not increased, activation of reward regions including the NAcc, caudate nucleus, and prefrontal areas compared with UD (Redlich et al., 2015). In another task study (Sharma et al., 2016), reduced activation in the bilateral VS and left OFC to social reward was found to be correlated with greater depression severity in the BD patients, but not the unipolar ones. These inconsistent findings may be explained by the variety of task paradigms. Accepting this, such different reward processing in BD and UD still suggests the role of the reward circuit in distinguishing the two disorders. Whether this different functioning of the reward circuit between BD and UD is state-dependent or has a trait-like profile is unclear. If recognized as a task-independent trait, differences of reward functioning should also exist at resting state, which may help to identify BD and UD earlier.

A resting-state fMRI study of the reward circuit can provide much benefit in the absence of specific tasks. During rs-fMRI scanning, participants are not required to perform a specific task. This avoids limitations due to the interference of different task paradigms and ensures a high degree of cooperation. Consequently, rs-fMRI may improve the relative consistency of findings across multiple studies. Previous studies suggested that resting-state functional connectivity (rsFC) between large-scale brain networks (Goya-Maldonado et al., 2016), and between region of interest (ROI) and other brain regions (Ambrosi et al., 2017), can differentiate unipolar and bipolar depression. One study exists which directly compared reward circuit rsFC between BD and UD (Satterthwaite et al., 2015). Moreover, aberrant reward circuit rsFC has already been identified in major depressive disorder (Felger et al., 2016; Gong et al., 2017) and many other medical conditions, including sleep disturbance (Avinun et al., 2017), attention deficit hyperactivity disorder (ADHD) (Dias et al., 2013; Tomasi and Volkow, 2014), and schizophrenia and cannabis use disorder (Fischer et al., 2014). This evidence verifies the dysfunction of the reward circuit not only during tasks but also at resting state.

In the present study, we aimed to explore neurobiomarkers for early recognition of BD by retrospectively comparing rsFC within the reward circuit between UD and depressed BD. Generally, the measure of rsFC can represent the functional synchronicity of spontaneous activity between a given region and any other regions in the whole brain. In this study, rsFC between key regions of the reward circuit (the OFC, the ACC, the VS, the VTA and the amygdala) was used to define functional synchronicity within the reward circuit. Interestingly, BD patients enrolled in our study were in depressive episodes at the baseline and had never experienced any manic/hypomanic episodes before. These depressed patients then began to manifest bipolar disorder (transformed bipolar disorder, tBD) during the follow-up period. We hypothesize that rsFC between key brain regions of the reward circuit differs between tBD and UD, which may contribute to the early distinction of BD from UD.

## Materials and Methods

### Participants

Seventy-seven patients with a preliminary diagnosis of MDD were enrolled at the Department of Psychiatry of the Affiliated Nanjing Brain Hospital of Nanjing Medical University from September 2011 to May 2017. The diagnosis of MDD was established according to the Diagnostic and Statistical Manual of Mental Disorders, fourth version (DSM-IV-TR). The 17-item Hamilton Rating Scale for Depression (HAMD-17) (Hamilton, 1960) was applied to assess depression severity. The Mini-International Neuropsychiatric Interview (M.I.N.I.) (Sheehan et al., 1998) was used to ensure the diagnosis of MDD and the absence of any other psychiatric disorders. The 32-item hypomania checklist (Hirschfeld et al., 2000; Angst et al., 2005) was used to screen out any lifetime manic/hypomanic episodes, with all patients scoring lower than 12. Participants with current or past history of other mental disorders were excluded.

The present study was a longitudinal observational follow-up study. During the follow-up period, patients were observed for at least 3 years unless they developed mania/hypomania. Patients who began to display mania/hypomania were defined as tBD. Thirty-seven patients were classified into the tBD group at the end of the observation in December 2017. The remaining 40 patients did not suffer from a manic/hypomanic episode after more than 3 years’ follow-up. It was considerable to refer to these patients as less likely to develop BD in the future, and they were defined as the UD group.

Forty healthy controls (HC) were recruited from local community. M.I.N.I. was also applied to confirm the absence of a psychosis history. HC were excluded if they reported family history of any mental disorders in first degree relatives.

All participants were Han Chinese, right handed, 18 to 55 years old, with a minimum education of 6 years. Additional exclusion criteria for all participants included nervous system disease, serious physical illness, substance abuse/dependence and any MRI contraindications.

This study was approved by the Research Ethics Review Board of Affiliated Nanjing Brain Hospital of Nanjing Medical University. All participants were informed of the study and provided written informed consent.

### MRI Data Acquisition

At the baseline after addition, all participants underwent MRI scan on a 3.0T Siemens Verio scanner with an 8-channel radio frequency coil at the Radiology Department of the Affiliated Nanjing Brain Hospital of Nanjing Medical University. Before the scan, subjects were instructed to lie still with their eyes closed, to relax but not fall asleep, and not to think of anything specific. Each subject was positioned comfortably in the coil and fitted with soft ear plugs to reduce scanner noise. Firstly, 3D T1-weighted images were acquired with the following parameters: repetition time (TR) = 1900 ms, echo time (TE) = 2.48 ms, flip angle = 9°, field of view (FOV) = 250 mm × 250 mm, matrix size = 256 × 256, 176 axial slices of 1 mm thickness, in-plane voxel resolution = 1 mm × 1 mm, acquisition time = 4 min 18 s. Further, a total number of 133 volumes of resting-state functional images (TR = 3000 ms, TE = 40 ms, flip angle = 15°, FOV = 240 mm × 240 mm, matrix size = 64 × 64, 32 axial slices of 4 mm thickness, acquisition time = 6 min 45 s) were acquired using gradient-recalled echo-planar imaging.

### Resting-State Functional Image Preprocessing

The image format was transferred using the MRIcroN^1^. Preprocessing was conducted by the Data Processing Assistant for Resting-State fMRI (DPARSF) toolbox^2^. First, the first 6 volumes were removed for stable magnetization and to adapt the participants to the scan. Then, the remaining 127 volumes were slice-time corrected, head-motion realigned, spatially normalized using a T1-weighted image by DARTEL segmentation, and saved with a spatial resolution of 2 mm × 2 mm × 2 mm. Smoothing was done with a 4-mm full-width at half maximum (FWHM) isotropic Gaussian kernel, temporal band pass filtering (0.01–0.08 Hz) was done to reduce low frequency drift and physiological high-frequency noise, and detrending was done to reduce the influence of the rising temperature of the MRI equipment. Subsequently, nuisance signals including Friston 24 head motion parameters as well as white matter and cerebrospinal signals were regressed out. In the present study, six patients (three UD and three tBD) and two HC were excluded due to head motion of more than 2.0 mm maximum displacement in any dimension or 2.0 degrees of angular motion. One HC was excluded for abnormal anatomic signals. Six participants (four UD, one tBD and one HC) were excluded because of bad normalization. Finally, 66 patients (33 UD and 33 tBD) and 36 HC went into further functional connectivity analysis in DPARSF.

### ROI-to-ROI Functional Connectivity Analysis

Based on the hypothesis mentioned above, we created 8 ROIs: the mOFC (MNI: 2, 46, -8), the ACC (MNI: -2, 28, 28), the left VS (MNI: -12, 12, -7) and the right VS (MNI: 12, 10, -6), the left Amy (MNI: -20, -2, -16) and the right Amy (MNI: 20, -2, -20), the left VTA (MNI: -4, -16, -14) and the right VTA (MNI: 4, -18, -14) at Montreal Neurological Institute (MNI) space. The coordinates of the mOFC, the ACC and the bilateral VS were derived from a meta-analysis of Bartra et al. (2013), which had been widely applied in fMRI studies (Satterthwaite et al., 2015; Pan et al., 2017). Concerning that Bartra’s meta-analysis didn’t provide precise coordinates of the amygdala, and that the sphere of the VTA in this meta-analysis may contain other structures of the brainstem, the coordinates of the bilateral Amy and the bilateral VTA were derived from an fMRI study that investigated the effects of city living on the reward system (Kramer et al., 2017). The WFU pickatlas^3^ was used to create ROIs with 4-mm-radius spheres for the bilateral VTA and 5-mm-radius spheres for the rest, centered according to previous studies (Kahn and Shohamy, 2013; Satterthwaite et al., 2015; Kramer et al., 2017; Pan et al., 2017). ROI-to-ROI functional connectivity was performed using the DPARSF toolbox. A time series of each ROI was extracted and averaged across all voxels within the ROI. Individual images were normalized into a standard template to get rid of individual location variance. Then, Pearson’s correlation coefficients between each pair of ROI regions were regarded as the strength of the functional connectivity. The correlation coefficients were transformed into Fisher’s z-score to improve normality and allow for further analysis. Thus, a *z*-score matrix of each individual functional connectivity was separately obtained. Since functional connectivity was directionless, we extracted the upper triangular matrix values (28 connections per subject) for statistical analysis.

### Statistical Analysis

One-way analysis of variance (ANOVA) and a chi-square test (only for gender) were performed in SPSS 19.0 software (SPSS Inc., Chicago, IL, United States) to compare demographic data. Clinical data which could be recorded as continuous variables including age at onset, total illness duration, current episode duration, and number of depressive episodes and HAMD-17 score between UD and tBD groups were analyzed using two-sample *t*-tests. Other clinical categorical variables were analyzed by chi-square tests between the two patient groups, such as family history of affective disorder, chronicity (defined as one single depressive episode that lasts for at least 2 years without significant remission) (Ratheesh et al., 2017), refractory (no improvement after sufficient treatment of two or more antidepressants), suicide attempt and diurnal depression variance, as well as treatment (type of antidepressant, stabilizer, rTMS, and MECT). Concerning that the treatment during the follow-up period might impact the prognosis of depression (i.e., whether they remain unipolar or develop into bipolar), binary logistic regression analysis was conducted. In the logistic regression analysis, the group (tBD or UD) was held as the dependent variable, and treatment was held as the independent variable. Significance was set at *P* < 0.05 two-tailed alternatives.

To examine the baseline rsFC differences between tBD and UD patients, a randomized permutation test with 5000 times was used. A permutation test is a type of statistical significance test in which the distribution of the test statistic under the null hypothesis is obtained by calculating all possible values of the test statistic under rearrangements of the labels on the observed data points (Nichols and Holmes, 2002). The detailed steps of the permutation test in this present study are as follows: (1) The averaged functional connectivity was computed for the original group labeling; (2) for each resampling, the group labels were randomly rearranged, and the averaged functional connectivity for the permuted data were computed; (3) step 2 was repeated until a predefined number of resamplings had been performed; and (4) the hypothesis was accepted or rejected based on the proportion of permuted averaged functional connectivity equal to or greater than the original. The rsFC differences in the baseline may serve as neurobiomarkers for early differentiation of BD from UD. As mentioned before, several clinical and demographic data were suggested to be risk factors for the transition from depression to BD, including family history of BD or affective disorder, early age of onset (less than 25 years-old), recurrence (more than four episodes), and refractory status (Dudek et al., 2013; Woo et al., 2015; Ratheesh et al., 2017). On the other hand, demographic factors such as age and education also might influence brain function. In order to test whether the between-group rsFC differences at baseline were due to these potential confounding factors, a general linear model (GLM) was performed. In the GLM, rsFC values of significant difference between UD and tBD were held as the dependent variables, groups were held as independent variables, and potential confounding factors (age, education years, onset age, number of episodes, family history, and refractory) were held as covariates. A significant difference was set at a threshold *P* < 0.05 FDR-corrected.

Additionally, to evaluate the accuracy of the rsFC values in predicting diagnosis (tBD vs. UD), we also carried out receiver operating characteristic (ROC) analysis, which could obtain the area under the curve (AUC) using SPSS software. Meanwhile, three statistics including sensitivity (SN), specificity (SP), and odds ratio (OR) were calculated to assess the diagnostic efficiency.

Our primary hypothesis concerned differences between tBD and UD patients. In order to provide information regarding the extent to which observed tBD and UD differences represent abnormal neural functioning, we also performed exploratory comparisons by including a group of HC and conducted permutation testing between HC and each patient group, respectively.

## Results

### Demographic and Clinical Characteristics

No significant differences in age, gender, and education level were found among the three groups. All clinical characteristics compared did not significantly differ between tBD and UD, including onset age, number of episodes, total illness duration, current episode duration, and total score of HAMD-17, family history of affective disorder, chronicity, refractory, suicide attempt, diurnal depression variance, and treatment (please see details in Table 1). Results of binary logistic regression analysis showed that group (tBD or UD) was not related to treatment (Supplementary Table 1), indicating that transition to BD was not due to differences in treatment.

**Table 1:** Demographic and clinical characteristics among three groups.

Variables	UD (*n* = 33)	tBD (*n* = 33)	HC (*n* = 36)	*P*-value
Sex (M/F)	17/16	17/16	18/18	0.941^a^
Age, y	30.91 ± 8.28	31.39 ± 8.30	32.41 ± 8.86	0.684^b^
Education, y	13.85 ± 3.02	13.88 ± 2.79	15.02 ± 2.26	0.053^b^
Onset age, y	28.03 ± 8.97	28.48 ± 9.29		0.840^c^
Total illness duration, mo	33.64 ± 53.31	40.50 ± 62.20		0.632^c^
Current episode duration, mo	7.55 ± 12.81	4.92 ± 4.97		0.279^c^
Number of episode	1.82 ± 1.26	2.09 ± 1.36		0.400^c^
Total score of HAMD-17	20.76 ± 9.26	22.27 ± 7.27		0.462^c^
Family history of AD	1 (3.0%)	4 (12.1%)		0.355^a^
Chronicity	5 (15.2%)	7 (21.2%)		0.751^a^
Refractory	3 (9.1%)	3 (9.1%)		1.000^a^
Suicide attempt	5 (15.2%)	11 (33.3%)		0.150^a^
Diurnal depression variance	15 (45.5%)	14 (42.4%)		1.000^a^
**Treatment**				
SSRI/SNRI	20/13	18/15		0.618^a^
Stabilizer	5 (15.2%)	9 (27.3%)		0.228^a^
rTMS	4 (12.1%)	4 (12.1%)		1.000^a^
MECT	4 (12.1%)	6 (18.2%)		0.733^a^

*UD, unipolar depression; tBD, transformed bipolar depression; HC, health control; HAMD, Hamilton Depression Rating Scale; AD, affective disorder; SSRI, selective serotonin reuptake inhibitor; SNRI, serotonin and norepinephrine reuptake inhibitor; rTMS, repetitive transcranial magnetic stimulation; MECT, modified electroconvulsive therapy.*

*^a^The P-value was obtained by two-tailed Pearson chi-square t-test.*

*^b^Data presented as the range of minimum-maximum (mean ± SD). The P-value was obtained by one-way analysis of variance.*

*^c^Data presented as the range of minimum-maximum (mean ± SD). The P-value was obtained by two-sample two-tailed t-test.*

### Resting-State Functional Connectivity

**FIGURE 1:**
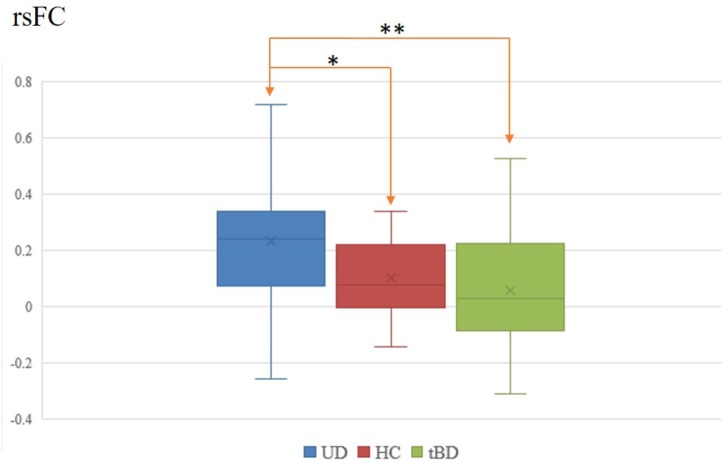
Left VTA and left VS rsFC difference between tBD, UD, and HC. ^∗^*P* < 0.05, uncorrected; ^∗∗^*P* < 0.05, FDR corrected.

**FIGURE 2:**
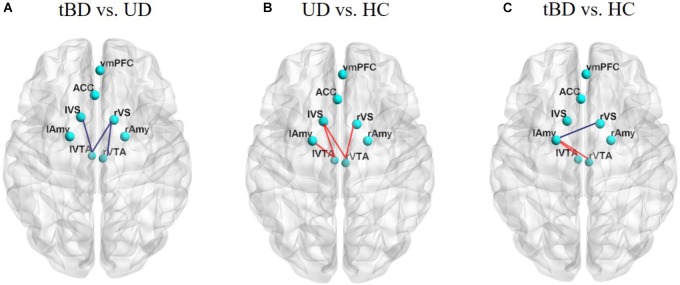
**(A)** Showed rsFC differences between tBD and UD. **(B)** Showed rsFC differences between UD and HC. **(C)** Showed rsFC differences between tBD and HC. The tBD showed significant lower rsFC between the left VTA and left VS (*P* = 0.001, *P* < 0.05 with FDR correction). Other rsFC differences were not significant (*P* < 0.05, uncorrected). rsFC presenting lower (blue) or higher (red). Superior view of a 3D brain. tBD, transformed bipolar disorder; UD, unipolar depression; mOFC, medial orbitofrontal cortex; ACC, anterior cingulate cortex; VS, ventral striatum; VTA, ventral tegmental area; Amy, amygdala.

Among the 28 connections within the reward circuit, rsFC between the left VTA and the left VS (rsFC value: tBD: 0.057 ± 0.223, UD: 0.234 ± 0.236; *P* = 0.001, *P* < 0.05 with FDR correction), between the left VTA and the right VS (rsFC value: tBD: 0.082 ± 0.223, UD: 0.234 ± 0.236; *P* = 0.008, uncorrected), and between the right VTA and right VS (rsFC value: tBD: 0.108 ± 0.229, UD: 0.227 ± 0.250; *P* = 0.049, uncorrected) were lower in the tBD group compared with the UD group. Only the rsFC between the left VTA and left VS was significantly different between tBD and UD (Figures 1, 2).

**FIGURE 3:**
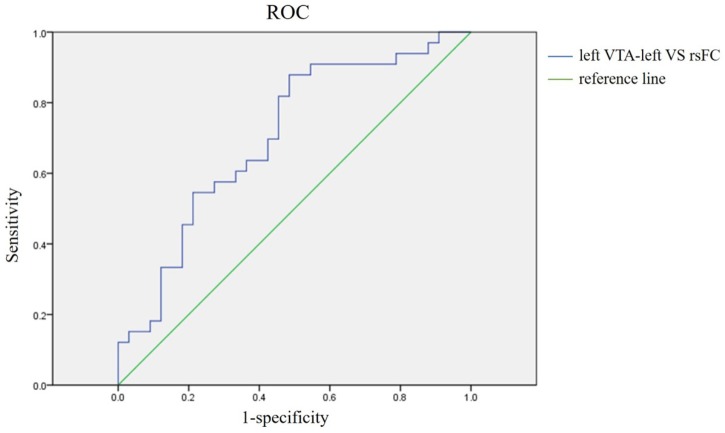
Receiver operating characteristic analysis of the left VTA-left VS rsFC. Area under curve (AUC) representing the sensitivity to the discriminant tBD and UD, AUC = 70%. Sensitivity = 87.9%, Specificity = 51.5%, odds ratio = 7.703, *P* = 0.005.

General linear model analysis showed that the rsFC differences between the two patient groups survived even after several possible confounding factors (family history of affective disorder, number of episodes, refractory, age of onset, education and age) were taken into account (Supplementary Table 2). Moreover, ROC analysis showed good accuracy of the left VTA-left VS rsFC (AUC = 70%, SN = 87.9%, SP = 51.5%, OR = 7.703, *P* = 0.005, Figure 3).

Exploratory comparisons showed that, relative to HC, UD showed higher rsFC between the left VTA and the left VS (rsFC value: UD: 0.234 ± 0.236, HC: 0.101 ± 0.137; *P* = 0.003, uncorrected), between the left VTA and the left Amy (rsFC value: UD: 0.271 ± 0.288, HC: 0.146 ± 0.267; *P* = 0.033, uncorrected), between the right VTA and the left VS (rsFC value: UD: 0.171 ± 0.228, HC: 0.024 ± 0.234; *P* = 0.005, uncorrected), and between the right VTA and the right VS (rsFC value: UD: 0.205 ± 0.243, HC: 0.077 ± 0.230; *P* = 0.015, uncorrected). Conversely, relative to HC, the tBD group showed higher rsFC between the bilateral VTA and the left Amy (left VTA-left Amy rsFC value: tBD: 0.270 ± 0.315, HC: 0.146 ± 0.267; *P* = 0.040, uncorrected. right VTA-left Amy rsFC value: tBD: 0.290 ± 0.301, HC: 0.126 ± 0.286; *P* = 0.012, uncorrected), and lower rsFC between the right VS and the left Amy (rsFC value: tBD: 0.227 ± 0.261, HC: 0.331 ± 0.226; *P* = 0.042, uncorrected). Unfortunately, these rsFC difference did not survive after FDR correction (Figures 1, 2).

## Discussion

This longitudinal study directly compared baseline rsFC within the reward circuit between tBD and UD. Consistent with our hypothesis, results showed that BD (in the depressive state before suffering from any mania/hypomania episodes) differed from UD in rsFC between the left VTA and the left VS. This result was not confounded by soft bipolar indications encompassing family history of affective disorder, refractory bipolar, or suicide attempt. The rsFC difference accounted for accurate differentiation between bipolar and UD in ROC analysis, which may contribute to the early distinction between the two affective disorders in depressive states.

Our main finding was that tBD showed lower VTA-VS rsFC compared with UD. The VTA and NAcc (main part of the VS) are key mesolimbic nodes in the reward circuit (Haber and Knutson, 2010), which are connected by the medial forebrain bundle (Keller et al., 2013). Dopamine projections from the VTA (site of dopamine neurons) to the NAcc represent the primary pathway in the reward circuit (Padoa-Schioppa and Cai, 2011; Russo and Nestler, 2013). Important aspects of reward processing are coded by dopaminergic neurons arising from the VTA and projecting to the ventral striatum (VS) via the mesolimbic pathway. The VTA–VS dopamine system has been found to be of eminent importance in a variety of motivated behaviors and cognition (Camara et al., 2009). VTA dopamine signals are suggested to modulate blood oxygenation level dependent (BOLD) signaling in the NAcc (Knutson and Gibbs, 2007), and is known to be crucial for reward processing (Padoa-Schioppa and Cai, 2011). Additionally, the VTA and the VS (NAcc) receive a multitude of afferents from cortical areas (medial prefrontal cortex, mOFC, dorsal ACC), limbic regions (Amy, hippocampus) and other brain regions implicated in reward processing (Camara et al., 2009; Russo and Nestler, 2013; Yetnikoff et al., 2014). Aberrant VS rsFC was suggested to reflect distributed striatal integration of coalescing signals from an impaired reward circuit (Pan et al., 2017). In our present study, resting-state functional synchronicity was different between patients in the prodromal phase of BD (tBD) and UD, but this difference was limited to the left VTA and left VS (center of the reward circuit). Meanwhile, no functional synchronicity differences were found in the rest of the reward circuit. This could explain why symptoms in depressive episode of tBD and UD were similar. On the other hand, reduced rsFC of the VTA–VS could be reflective of an impaired dopamine signaling system. Therefore, lower rsFC of VTA-VS may indicate that depression related to BD may be more severe than that of UD. The rsFC differences between the left VTA and the left VS in our study possibly indicate divergent dysfunction in the reward circuit.

Numerous task-related fMRI studies have verified reward circuit dysfunction of hyperactivation (or hyperconnectivity) in BD (Nusslock et al., 2012; Schreiter et al., 2016) and hypoactivation (or hypoconnectivity) in UD (Ubl et al., 2015; Gong et al., 2017), respectively. The only study directly investigating rsFC within the reward circuit between BD and UD, to our knowledge, reported higher functional connectivity at resting state within the reward system, including the VS, VTA, anterior insula and thalamus in BD compared with UD (Satterthwaite et al., 2015). These are contrary to our present results of lower VTA-VS rsFC. Such a discrepancy could due to the fact that patients labeled as BD in Satterthwaite’s study already experienced mania/hypomania before fMRI scanning, unlike our prodromal “bipolar” depressive ones. Similarly, the abovementioned study failed to find any significant differences of rsFC within the reward circuit between BD and HC as we did in the present study. Although speculative, it is possible that functional synchronicity of the reward circuit at rest is normal in depressive episodes, but already different between patients in the prodromal phase of BD and those with UD. As diagnosis of BD is precisely established at the onset of mania, the reward circuit is impaired severely enough and overreacts to reward stimuli during tasks (Alloy et al., 2015). There is lower functional synchronicity within the reward circuit at rest before mania but higher during tasks in BD vs. UD; such low-to-high fluctuation prompts more serious impairments in BD, which could be supported by the evidence of reward hypersensitivity in BD and hyposensitivity in UD (Alloy et al., 2016).

Notably, differences in rsFC within the reward circuit were only demonstrated between the VTA and the VS, which could be explained by several reasons. On one hand, the VTA-VS system, as an essential pathway in the reward circuit (Russo and Nestler, 2013), might be the first or optimal feature for the early distinction of bipolar from unipolar. On the other hand, the current methodology of fMRI rsFC is useful but maybe not powerful enough to detect other identification, which will be achieved by future developments in neuroimaging.

### Limitation

Some limitations should be considered. First, UD patients enrolled in this study still have the possibility to manifest BD in the future (Ratheesh et al., 2017). In light of this point, grouping is not absolutely correct. To reduce this potential impact, the diagnoses of MDD in the UD group were confirmed by a follow-up lasting of no less than 3 years. Such a follow-up design and the transition rate (8.6∼25%) (Holma et al., 2008; Gilman et al., 2012; Woo et al., 2015; Bukh et al., 2016; Holmskov et al., 2017; Ratheesh et al., 2017) limited the sample size of UD and tBD, respectively. Secondly, an additional resting-state fMRI scan at the end of the follow-up, especially for tBD, may replicate previously consistent findings (reward hyperactivity in BD and hypoactivity in UD) (Alloy et al., 2016), which would make our conclusion more reliable. Although in the absence of such additional data due to retrospective design, our findings did expand the knowledge of BD in the prodromal stage. Lastly, the measure of functional connectivity fails to illustrate the direction of abnormal brain interaction due to the relatively low time resolution of rs-fMRI data (common in most other rs-fMRI studies). Developing more advanced neuroimaging techniques may help overcome this disadvantage in future.

## Conclusion

In conclusion, the present study verified the hypothesis that bipolar disorder, in its prodromal stage of mania/hypomania, differs from UD in the reward circuit of VTA-VS functional synchronicity during resting-state, which exhibited good accuracy for early distinction between the two mood disorders. Our findings, together with the previous reward hypersensitivity theory, might indirectly indicate more severe impairment of the reward circuit in bipolar disorder.

## Author Contributions

ZY and QL designed the experiments. JS and JG performed the experiments. JS wrote the manuscript. JG, RY, XL, and RZ contributed to clinical data collection and assessment. JG, XW, JS, MX, and KB analyzed the results. ZY, QL, and JS approved the final manuscript. All authors assisted with carrying out the experiments.

## Conflict of Interest Statement

The authors declare that the research was conducted in the absence of any commercial or financial relationships that could be construed as a potential conflict of interest.

## Footnotes

1 http://www.mricro.com

2 http://www.restfmri.net/forum/DPARSF

3 http://fmri.wfubmc.edu/software/pickatlas
