# Supplementary material for: Differentiation of Transformed Bipolar Disorder From Unipolar Depression by Resting-State Functional Connectivity Within Reward Circuit
Source: Front Psychol. 2018 Dec 21;9:2586. doi: 10.3389/fpsyg.2018.02586 (PMC6308204; doi:10.3389/fpsyg.2018.02586)
Supplement: Supplementary file 1 [file Data_Sheet_1.docx]

Supplementary Table 1. Binary logistic regression analysis between group and treatment

| Treatment | B value | *P*-value | 95% C.I. of Exp(B) |
| --- | --- | --- | --- |
| SSRI/SNRI | -0.276 | 0.599 | 0.270-2.128 |
| stabilizer | -0.746 | 0.238 | 0.137-1.636 |
| rTMS | -0.039 | 0.961 | 0.201-4.613 |
| MECT | -0.398 | 0.582 | 0.163-2.768 |

Supplementary Table 2. GLM between rsFC and possible confounding factors

| Factors | F value | *P*-value |
| --- | --- | --- |
| Age | 0.002 | 0.961 |
| Education | 1.146 | 0.289 |
| Onset age | 0.178 | 0.675 |
| Number of episodes | 0.263 | 0.610 |
| Family history | 0.231 | 0.632 |
| Refractory | 0.679 | 0.413 |
